# Supplementary material for: Fiber-optic seismic sensing of vadose zone soil moisture dynamics
Source: Nat Commun. 2024 Aug 5;15:6432. doi: 10.1038/s41467-024-50690-6 (PMC11300608; doi:10.1038/s41467-024-50690-6)
Supplement: Supplementary file 1 — Supplementary Information [file 41467_2024_50690_MOESM1_ESM.pdf]

Supplementary Materials for

Fiber-optic seismic sensing of vadose zone soil moisture dynamics

Zhichao Shen[1,2,5], Yan Yang[1,5], Xiaojing Fu[3], Kyra H. Adams[4], Ettore Biondi[1], Zhongwen Zhan[1]

Corresponding e-mail: zhichao.shen@whoi.edu

The PDF file includes:

Supplementary Figures 1-7
Supplementary Tables 1-2

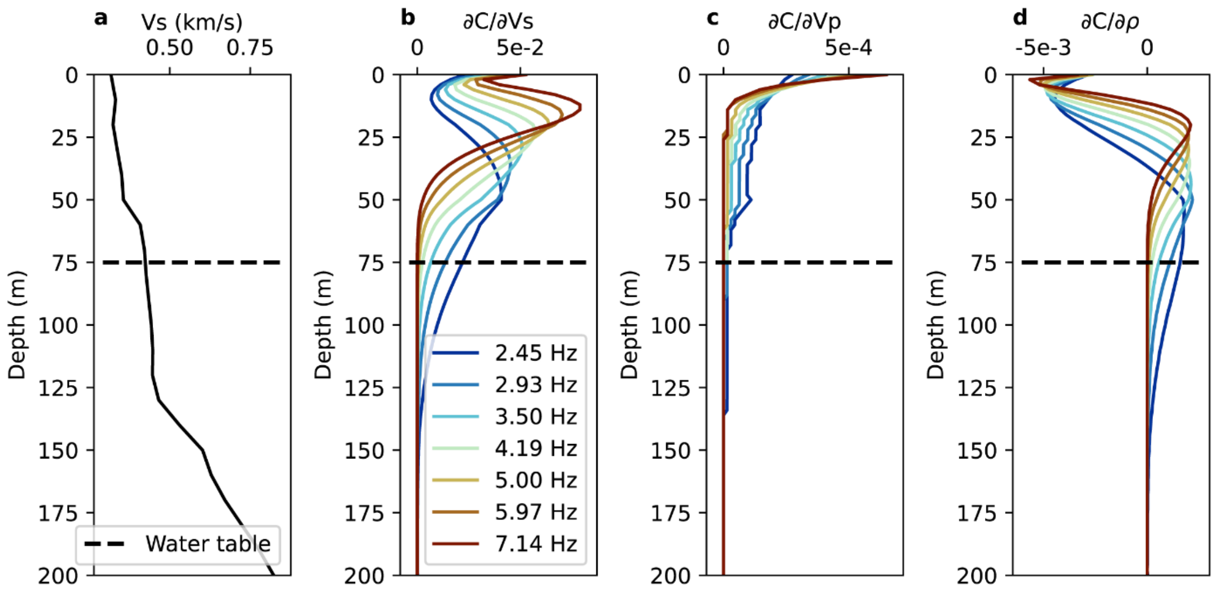

Supplementary Fig. 1. Shear velocity model and surface wave sensitivity kernels. (a) Shear velocity model averaged along the cable. The sensitivity kernels of frequency-dependent surface wave velocity with respect to depth-dependent (b) shear velocity, (c) compressional velocity, and (d) density. Black dashed lines represent the local groundwater level. For all the sensitivity kernels, the measured dv/v that monotonically increases with frequency should be sensitive to the vadose zone above 20-m depth rather than groundwater.

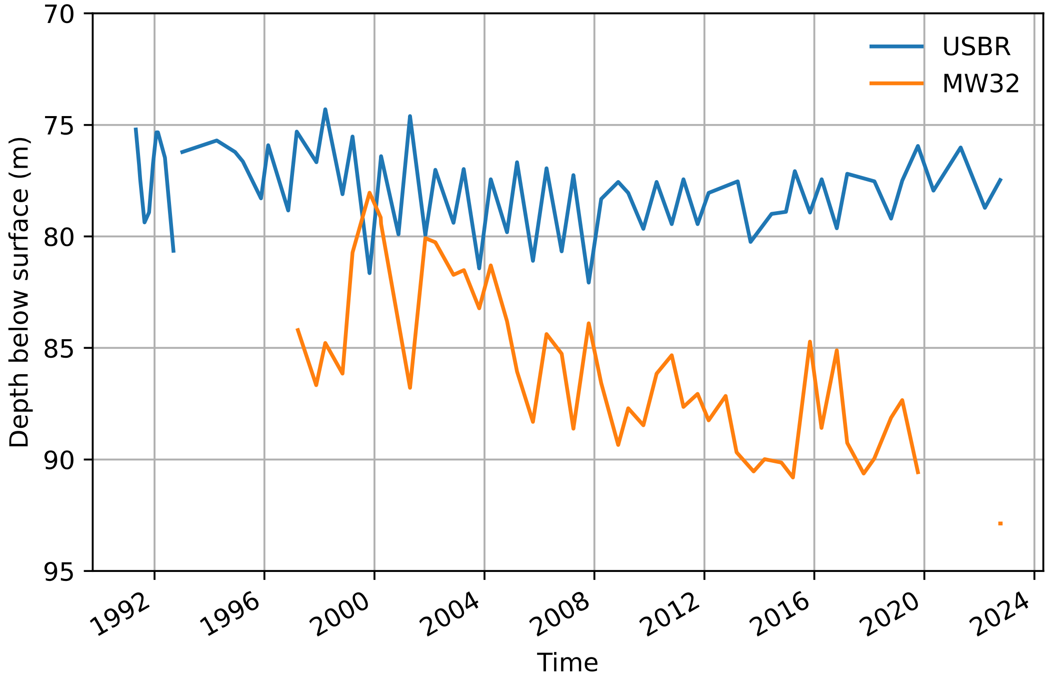

Supplementary Fig. 2. Groundwater level data in the past twenty years measured at nearby groundwater monitoring wells USBR and MW32. The locations of the two wells are indicated in Fig. 1.

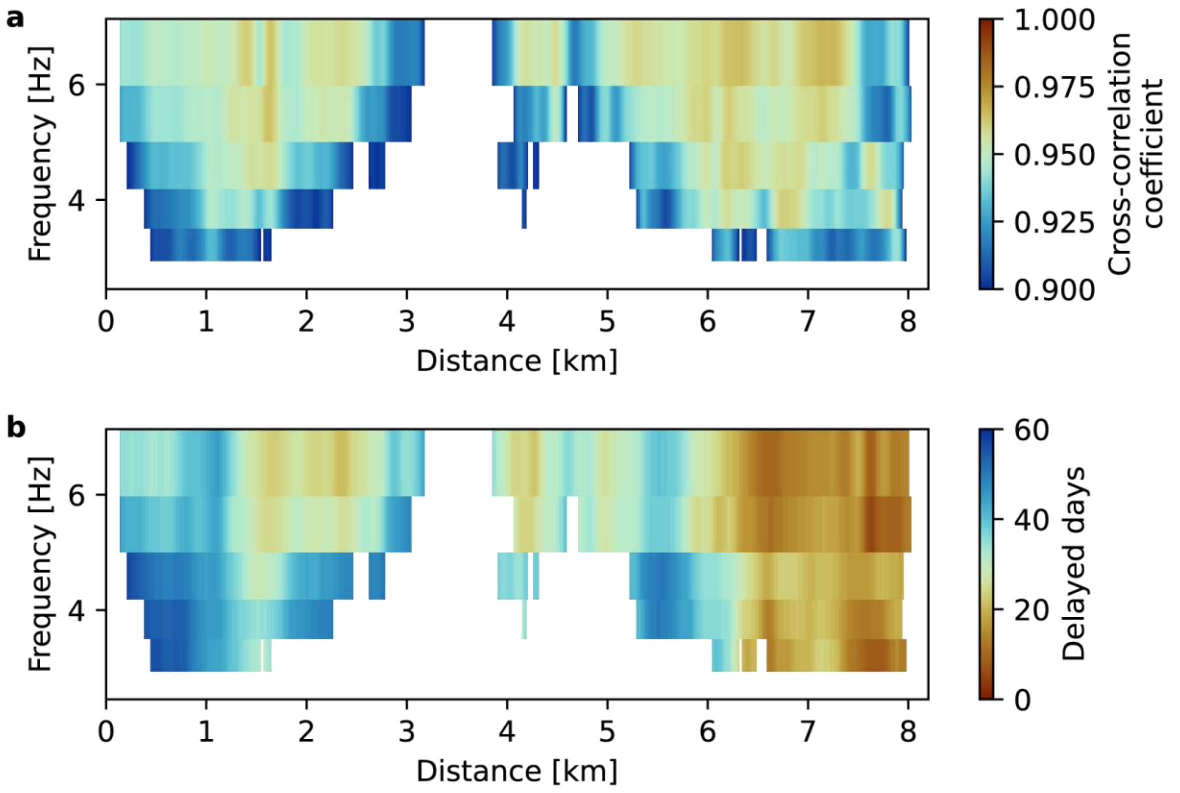

Supplementary Fig. 3. Fitting dv/v with 60-day smoothed surface temperature for all the channels and frequencies. (a) maximum correlation coefficient. (b) best fitted time delay. The correlation coefficient between dv/v and temperature increases with frequency, whereas the phase-lag decreases with frequency.

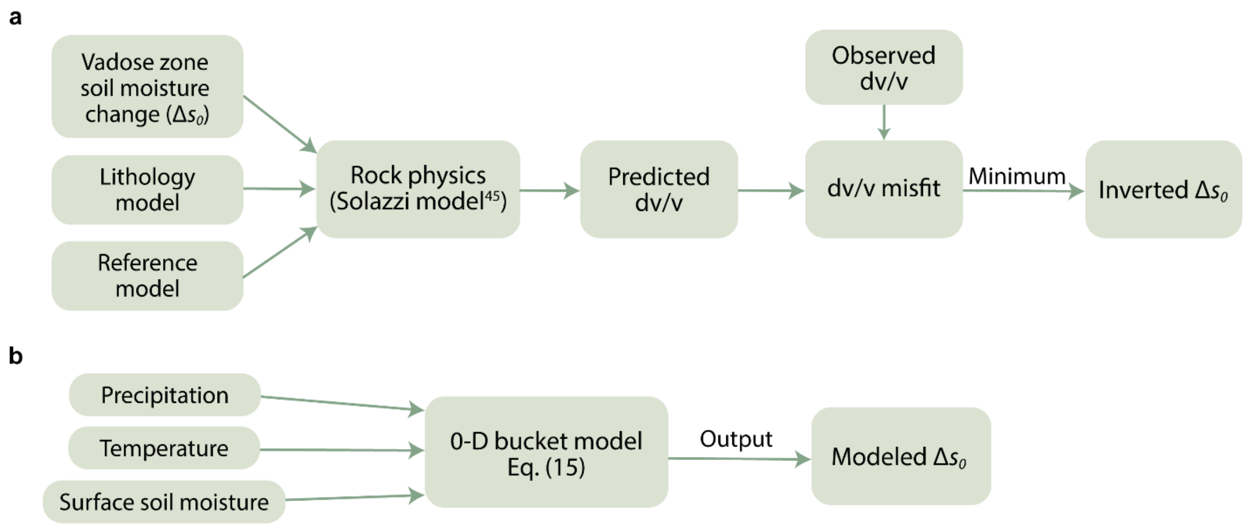

Supplementary Fig. 4. Flowchart describing major steps in obtaining inverted and modeled vadose zone soil moisture changes. (a). Major steps obtaining inverted daily soil moisture changes from dv/v observations. (b). Major steps in our hydrological modeling.

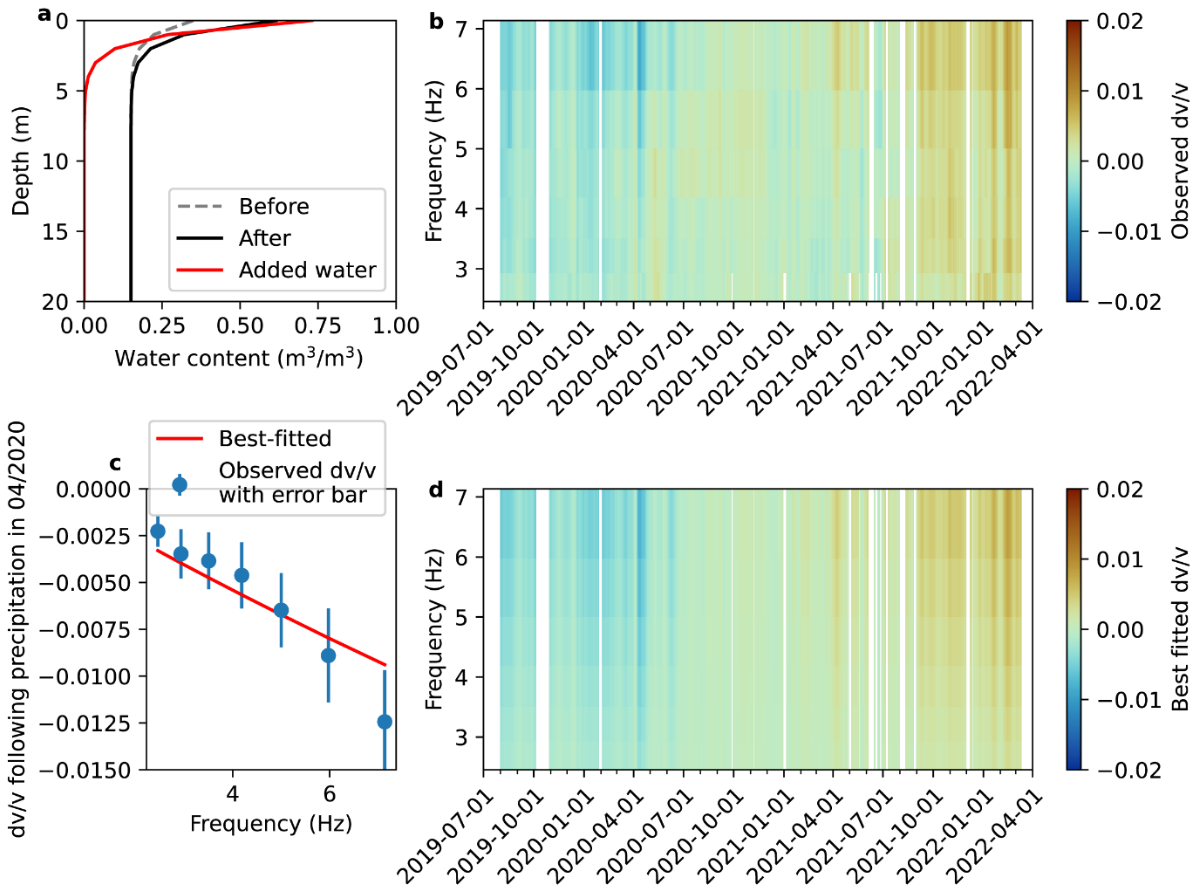

Supplementary Fig. 5. Inversion from dv/v to average water content changes in the vadose zone. (a) This panel displays the reference (gray dashed line), added (red line), and resultant (black line) depth profiles of water content within the top 20 meters of the vadose zone. (b) Over a 2.5-year period and across 7 frequency bands, the dv/v measured by DAS is averaged for a cable segment between 7.2 and 7.6 km along the cable. (c) The observed dv/v values for the day after the significant precipitation event in April 2020 are plotted as blue dots with error bars. The error bar is given as the variability of dv/v measurements along the cable segment (i.e., 7.2-7.6 km). The red line represents the synthetic dv/v computed using the water profile from panel (a). (d) Synthetic dv/v computed from the best-fitted inversion model, which is shown in the red curve of Fig. 4.

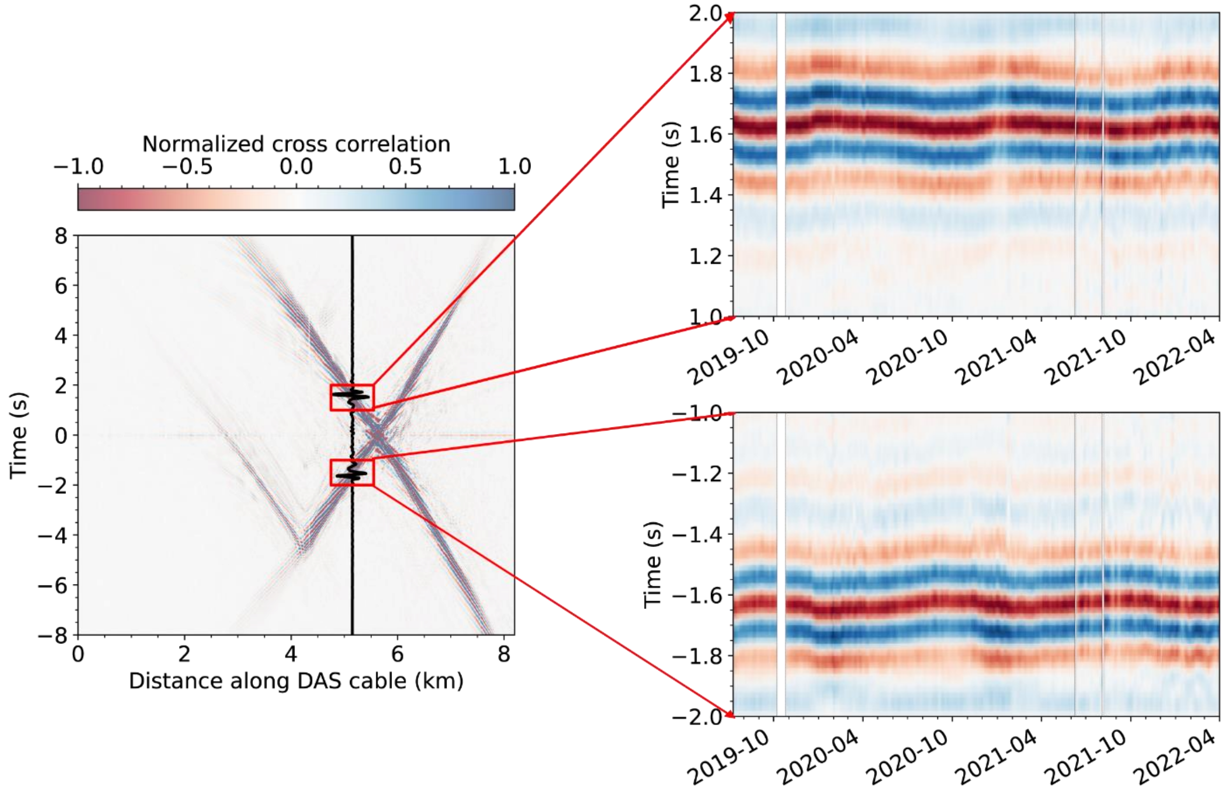

Supplementary Fig. 6. Example of direct Rayleigh waves on cross-correlation waveform. The symmetry of the Rayleigh waves indicates that the source distribution is generally uniform in space, and the resulting dv/v is attributed to the medium rather than noise source variations.

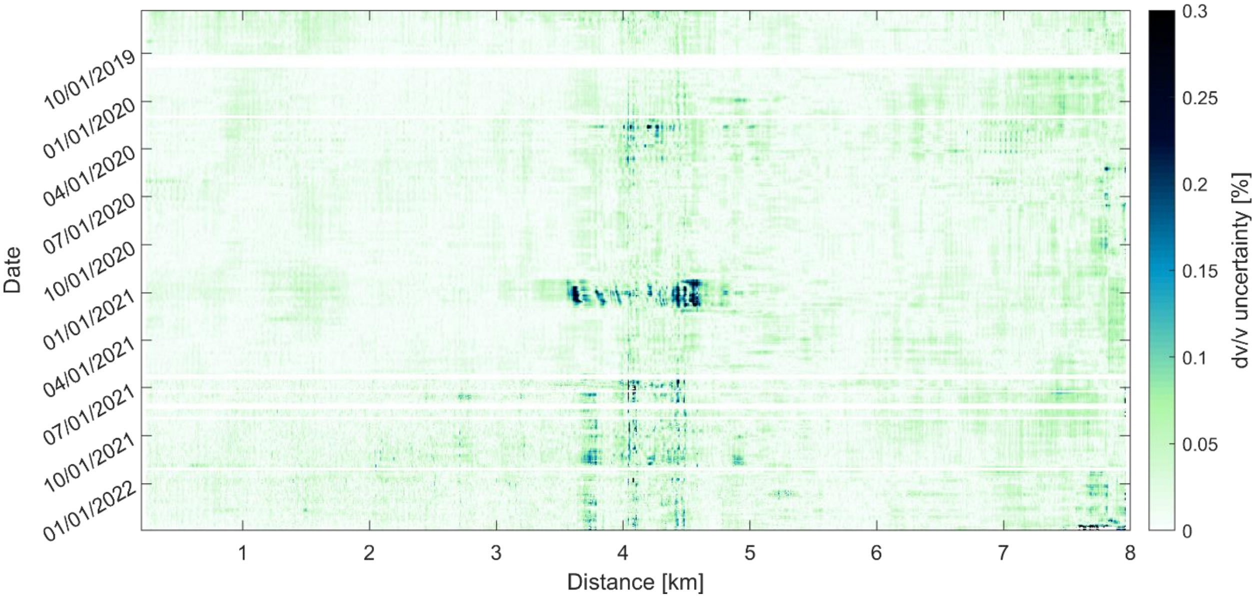

Supplementary Fig. 7. dv/v measurement uncertainty for the central frequency of 4.2 Hz. Note the relatively large uncertainty near 4 km (vertical stripes) due to the scattered surface waves in Supplementary Fig. 6.

Year	Jan	Feb	Mar	Apr	May	Jun	Jul	Aug	Sep	Oct	Nov	Dec
**2019**	0	0	44.20	7.37	0	0	0	0	0	0	13.21	0
**2020**	0	1.78	23.11	48.00	0	0	0	0	0	0	0	0
**2021**	2.03	0	0	0	0	0	6.86	0	1.27	1.27	0	27.18
**2022**	0	0	0	0	0	2.79	0	8.13	0	0	0	8.13

*Shaded area represents our dv/v data coverage.

Supplementary Table 1. Monthly Total Precipitation (mm) for meteorological station NID

Soil Type	Soil Porosity (ϕ)	Average number of contacts per particle (N)	Fraction of non-slipping particles (f)	Quartz (%)	Kaolinite (%)
**Esperance sand**	0.25	6	0.1	70	30
**Missouri clay**	0.25	8	0.3	10	90

Supplementary Table 2. Properties and components of endmember soils.
